# PD-L1 expression, morphology, and molecular characteristic of a subset of aggressive uterine tumor resembling ovarian sex cord tumor and a literature review

**DOI:** 10.1186/s13048-023-01183-5

**Published:** 2023-05-23

**Authors:** Si-Ping Xiong, Rong-Zhen Luo, Fang Wang, Xia Yang, Jun-Peng Lai, Chao Zhang, Li-Li Liu

**Affiliations:** 1 State Key Laboratory of Oncology in South China, Collaborative Innovation Center for Cancer Medicine, Sun Yat-sen University Cancer Center, Guangzhou, 510060 China; 2 Department of Pathology, Sun Yat-sen University Cancer Center, 651# Dong Feng Road East, Guangzhou, 510060 China; 3 Department of Pathology, The Eighth Affiliated Hospital of Sun Yat-sen University, Shenzhen, Guangdong 518033 China; 4 Department of Molecular Diagnostics, Sun Yat-Sen University Cancer Center, Guangzhou, 510060 China

**Keywords:** Uterine tumors resembling ovarian sex cord tumor, Mitotic activity, PD-L1, NCOA2

## Abstract

**Background:**

Uterine tumors resembling ovarian sex cord tumor (UTROSCT) is a rare neoplasm of unknown etiology and has undetermined malignant potential. The emergence of recurrent UTROSCT case reports has led to its initial identification as a tumor of low malignancy potential. Owing to its low incidence, we currently lack any in-depth studies regarding the subset of UTROSCTs that may be aggressive in nature. Here, we sought to identify unique characteristics in aggressive UTROSCT.

**Methods:**

19 cases of UTROSCT were collected. Their histologic and tumor immune microenvironment were evaluated by three gynecologic pathologists. The gene alteration was also detected by RNA sequencing. For later analyses regarding differences between benign and malignant tumors, we supplemented our 19 included cases with additional reports from the literature.

**Results:**

Interestingly, we found PD-L1 expression in stromal tumor-infiltrating immune cells (stromal PD-L1) was markedly higher in aggressive UTROSCT. Patients with high stromal PD-L1 (≥ 22.5 cells/mm^2^) had worse prognosis. When our cases were added with previous cases identified in the literature, we discovered that aggressive UTROSCT was more likely to have significant mitotic activity and NCOA2 gene alterations than benign UTROSCT. Consistence with those results, patients with significant mitotic activity and gene alteration of NCOA2 had worse prognoses.

**Conclusions:**

Collectively, high expression of stromal PD-L1, significant mitotic activity, and gene alteration of NCOA2 may be useful markers to predict aggressive UTROSCT.

**Supplementary Information:**

The online version contains supplementary material available at 10.1186/s13048-023-01183-5.

## Introduction

Uterine tumors resembling ovarian sex cord tumor (UTROSCT) is a rare neoplasm of unknown etiology and has undetermined malignant potential [1]. Histologically, UTROSCT presents as mainly sex cord like differentiation with various morphological appearances and many phenotypic immunoprofiles [2]. Dating back to 1945, Morehead and Bowman were the first to report uterine tumors with sex cord-like differentiation [3]. Then, in 1976, Clement and Scully divided these tumors into two groups based on the clinical and histopathological characteristics of 14 cases [4]. After this classification, two types emerged: (1) Endometrial stromal tumors with focal sex cord differentiation (<50% of the mass) were identified as endometrial stromal tumors with sex cord-like elements (ESTSCLEs) and (2) tumors containing predominantly and/or exclusively sex cord differentiation without stromal component were identified as UTROSCT [4]. The current World Health Organization (WHO) classification includes UTROSCT under the category of “Miscellaneous mesenchymal tumors”, and defines it as a uterine neoplasm with a morphological pattern that resembles those seen in ovarian sex cord tumors, without a component recognizable as endometrial stroma [5].

Given advancements in sequencing and biomarker identification, next-generation sequencing (NGS) and RNA-seq have resulted in the identification of several molecular pathogeneses leading to the developing of UTROSCT, including rearrangements of either estrogen receptor 1 (ESR1) or growth regulating estrogen receptor binding 1 (GREB1). Their molecular partners are always those of the nuclear receptor coactivator (NCOA) family, including NCOA1, NCOA2, and NCOA3 [2, 6]. UTROSCT also lacks the molecular characteristics of adult granulosa cell tumor (FOXL2 mutations), Sertoli-Leydig cell tumor (DICER1 mutations), or low-grade endometrial stromal sarcoma (JAZF1-SUZ12 gene fusion) [7–9].

Clinically, UTROSCTs were initially identified as benign tumors. However, the emergence of recurrent UTROSCT case reports has resulted in reconsideration of UTROSCT as a tumor with low malignancy potential. In Blake and his colleague’s study, the recurrence rate identified was nearly 5.5% [10]. Recent work by Michelle and colleagues indicated that this rate had reached to 23.5% [11]. However, given the low incidence of UTROSCT, the clinical and histological characteristic of malignant UTROSCT remain unknown. Therefore, further investigation is needed to classify the subset of aggressive uterine tumor resembling ovarian sex cord tumors. In our study, we elucidated the histopathology of aggressive UTROSCT, including its morphology, tumor microenvironment, and molecular characteristic.

## Materials and methods

### Patients and samples

Our study was approved by the Institutional Ethical Boards of Sun Yat-sen University Cancer Center. Nineteen cases of UTROSCT were collected. The study methodologies conformed to the standards set by the Declaration of Helsinki. Histologic and immunohistochemical slides of the collected cases were reviewed by three gynecologic pathologists who confirmed diagnosis of UTROSCT in all cases. Further information regarding the clinical, histologic, and molecular characteristics of these UTROSCT cases are displayed in Table 1. One case was identified as having a JAZF1-SUZ12 gene fusion and was excluded for further study. For later analyses regarding differences between benign and malignant tumors, we supplemented our 19 included cases with additional reports from the literature.

### Immunohistochemistry

To diagnosis UTROSCT, immunochemistry was used in conjunction with a series of antibodies, including inhibin α, calretinin, WT1, CD56, desmin and etc. Detailed information regarding the antibodies used is shown in Supplementary Table 1. For our tumor microenvironment analysis, PD-L1, FOXP3, and CD8 antibodies were also used. Detailed information regarding these antibodies is also shown in Supplementary Table 1.

Tumoral PD-L1, PD-L1 in stromal tumor-infiltrating immune cells (stromal PD-L1), CD8 + and FoxP3 + T lymphocytes were manually enumerated by 2 pathologist (Lili Liu and Siping Xiong) and averaged to per mm^2^ [12]. For tumoral PD-L1, the PD-L1 expressed in tumor cells with any intensity of membrane expression were evaluated. For stromal PD-L1, we calculated in both lymphocytes and macrophages which were directly in contact with the tumor (either intratumoral or in direct contact with the tumor periphery) [12].

### RNA sequencing

RNA sequencing (RNA-Seq) was convicted as previously described [1]. Briefly, RNA was extracted from formalin-fixed paraffin-embedded tissue using the commercially available ExpressArt FFPE Clear RNA Ready kit (Amsbio, Cambridge, MA) and according to the manufacturer’s instructions. After quantifying the total RNA and preparing RNA-Seq libraries, RNA samples (20–100 ng) was used in all later experiments. Each library was sequenced using the TruSight RNA Fusion Panel (Illumina, SanDiego, CA).

### Statistical analysis

Statistical analyses were performed using SPSS 26.0 (SPSS, Chicago, IL, USA). Student’s t test was used to assess differences in our tumor microenvironment assay; Chi-squared test was used to analyze the correlations between malignancy and histopathology features as well as molecular characteristics. Kappa Meier analysis was used for prognosis analysis.

## Results

### Clinical, morphologic, and molecular features

In this study, we used morphologic and immunohistochemical characteristics to diagnose 19 cases. Their clinical, morphological, and molecular features are shown in Table 1. The mean age of patients was 42.8 years with a range of 19–58 years. Eleven patients (57.9%, 11/19) received a hysterectomy. The mean tumor size was 4.1 cm (range, 1.5–15 cm). The median follow-up was 40.9 months (range, 1.2-195.3 months). Of the included cases, 6 patients (31.6%, 6/19) had a tumor recurrence and the clinical details of these malignant cases are summarized in Supplementary Table 2. The other 13 patients remain alive without evidence of recurrent disease.

**Table 1:** Clinical, histologic, and molecular characteristics of UTROSCT (n = 19)

Case	Age (y)	Surgery	TS (cm)	Tumor Margins	Tumor architecture	Cyto- morphology	Necrosis	Significant nuclear atypia	Mitoses (/10HPF)	NCOA1-3 Fusion	NCOA1-3 Fusion Detection Method	JAZF1/SUZ12/ PHF1 Rearrangement	Follow-up
1	31	NA	2.5	Infiltrative	Sertoliform	Spindle and epithelioid	No	Yes	< 1	ESR1-NCOA2	RNA-Seq	Negative	NTR (18.9 m)
2	35	NA	3	Infiltrative	Sertoliform, corded	Spindle and epithelioid	No	No	< 1	NA	NA	NA	NTR (40.9 m)
3	51	NA	3	Infiltrative	Sertoliform, nested	Spindle and epithelioid	No	No	< 1	NA	NA	NA	NTR (43.9 m)
4	41	Yes	5.5	Infiltrative	Sertoliform, nested, corded	Spindle and epithelioid	No	No	3	ESR1-NCOA2	RNA-Seq	Negative	Metastasis (144.4 m)
5	53	Yes	3	Infiltrative	Sertoliform, trabecular, retifrom	Spindle and epithelioid	No	No	2	GREB1-NCOA2	RNA-Seq	Negative	NTR (44.7 m)
6	33	NA	3	Infiltrative	Sertoliform, corded	Spindle and epithelioid	No	No	2	ESR1-NCOA3	RNA-Seq	Negative	NTR (54.9 m)
7	48	Yes	2	Infiltrative	Nested	Spindle and epithelioid	No	No	2	NA	NA	NA	NTR (56.4 m)
8	44	NA	3	NA	Sertoliform, corded	Spindle and epithelioid	NA	NA	NA	NA	NA	NA	NTR (62.3 m)
9	46	NA	2.5	Infiltrative	Sertoliform, retiform	Spindle and epithelioid	No	No	2	Negative	RNA-Seq	Negative	Died (26.3 m) Metastasis (2.5 m)
10	19	NA	3	Infiltrative	Corded, trabecular	Spindle and epithelioid	No	No	2	NA	NA	NA	Metastasis (69.9 n)
*11	52	Yes	15	Circumscribed	Corded, trabecular	Epithelioid	Yes	Yes	8	Negative	RNA-Seq	JAZF1-SUZ12	NTR (14.0 m)
12	58	Yes	4	Infiltrative	Sertoliform, retiform	Spindle and epithelioid	No	Yes	2	GREB1-NCOA1	RNA-Seq	Negative	NTR (22.6 m)
13	36	Yes	1.5	Infiltrative	Sertoliform, retiform	Spindle and epithelioid	Yes	Yes	10	Negative	RNA-Seq	Negative	Metastasis (56.5 m)
14	55	Yes	13	Infiltrative	Sertoliform, corded, trabecular	Spindle and epithelioid	No	Yes	2	GREB1-NCOA2	RNA-Seq	Negative	Metastasis (195.3 m)
15	48	Yes	3	Infiltrative	Sertoliform, retiform	Spindle and epithelioid	No	Yes	< 1	ESR1-NCOA3	RNA-Seq	Negative	NTR (9.2 m)
16	44	No	3	Infiltrative	Sertoliform, corded, trabecular	Spindle and epithelioid	Yes	Yes	2	Negative	RNA-Seq	Negative	NTR (1.2 m)
17	29	Yes	3	Circumscribed	nested	Spindle and epithelioid	Yes	Yes	8	Negative	RNA-Seq	Negative	NTR (7.9 m)
18	42	Yes	3.3	Circumscribed	Sertoliform, retiform	Spindle and epithelioid	No	Yes	< 1	NA	NA	NA	NTR (9.2 m)
19	48	Yes	2.2	Infiltrative	Sertoliform, retiform	Spindle and epithelioid	NA	NA	NA	NA	NA	NA	Metastasis (21.1 m)

TS, tumor size; NA, not available; NTR, no tumor recurrence

^*^Excluded from statistics due to molecular translocation suggesting an endometrial stromal neoplasm (JAZF1-SUZ12)

**Fig. 1:**
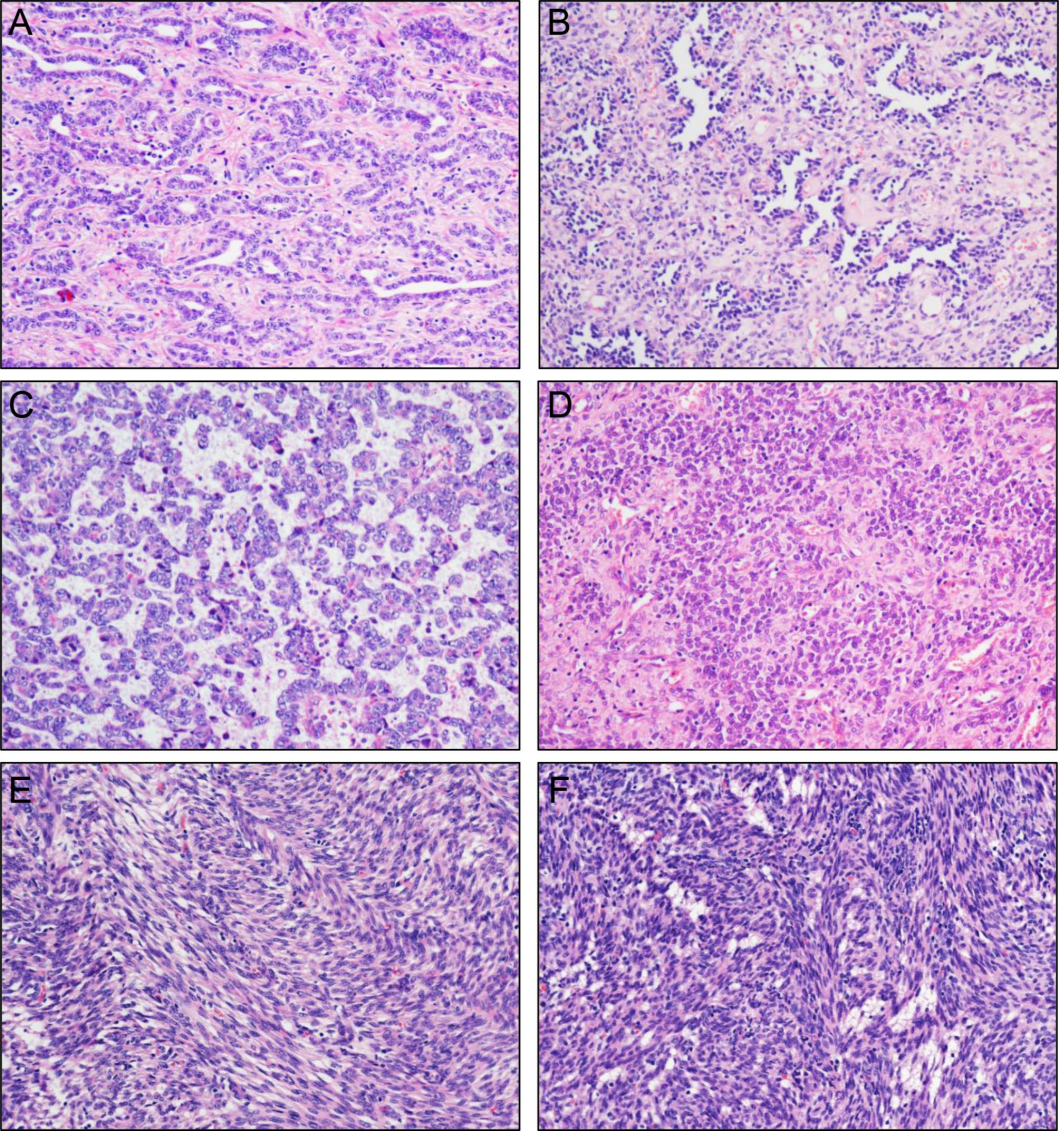
**Various morphologic patterns in UTROSCT.** A, Tubules lined by epithelioid cells with scant cytoplasm. B, Tumor centered in myometrium with a retiform pattern of epithelioid cells, and area of interstitial hyalinization. C, Tumor cells in the form of nested and cord patterns. D, Nests of plump epithelioid cells with abundant eosinophilic cytoplasm. E, Sheets of spindle cells. F, Aggregates of cells with foamy cytoplasm

Most of the cases showed infiltrative tumor margins and only three patients had circumscribed tumor margins. All cases with different architectural appearance contained predominantly sex cord differentiation. The morphologic patterns of sex cord differentiation contained corded, trabecular, sertoliform, retiform, and etc. (Fig. 1). No lymphovascular invasion was observed. The percentages of significant mitotic activity, significant nuclear atypia, and necrosis in tumors were as follows: 63.2% (12/19), 47.4% (9/19), and 21.1% (4/19), respectively.

Using RNA-seq, gene alterations were identified in 8 cases. GREB1-rearranged tumors were identified in three cases; similarly, ESR1-rearranged tumors were identified in 4 cases. We also identified one tumor with a JAZF1-SUZ12 fusion. The clinical and morphologic characteristics of the GREB1- and ESR1-rearranged tumors are presented in Supplementary Table 3. A detailed diagrammatic sketch of RNA-seq result for a malignant case (Case 4) is shown in Figure S1. Case 11 contained the JAZF1-SUZ12 fusion, which suggested endometrial stromal sarcoma. Given this, we excluded Case 11 from any further analysis.

### Immunohistochemical features

The immunohistochemical profile of all cases is shown in Table 2. UTROSCT is a polyphenotypic neoplasm that expresses epithelial, sex cord, and smooth muscle markers, as well as hormone receptors. Possible sex cord markers include inhibin α, calretinin, WT-1, CD56, and CD99. Of the included cases, 17 (17/19, 89.5%) were positive for at least one of these markers. The remaining two cases that were negative for sex cord markers were Cases 7 and 11. However, WT-1 and CD56 were not available and we were unable to assess for any additional sex cord markers.

**Table 2:** Immunohistochemical features of cases (n = 19)

Case	1	2	3	4	5	6	7	8	9	10	^*^11	12	13	14	15	16	17	18	19
**Inhibin**	-	-	+	-	+	-	-	-	+	-	-	+	-	-	-	-	-	-	-
**Calretinin**	+	NA	NA	NA	-	-	-	-	-	-	-	+	NA	-	NA	+	-	-	NA
**WT1**	+	+	+	+	NA	NA	NA	+	NA	NA	NA	+	NA	+	NA	+	NA	+	+
**CD56**	+	+	+	NA	+	+	NA	+	NA	NA	NA	+	+	+	+	+	NA	+	+
**AE1/AE3**	+	-	+	+	+	+	+	NA	+	+	+	+	+	-	+	-	+	-	-
**Desmin**	-	+	+	+	-	+	+	+	-	-	+	+	NA	-	-	+	+	NA	+
**SMA**	-	NA	+	-	-	NA	+	+	-	-	+	+	-	NA	NA	-	+	+	-
**Caldesmon**	NA	NA	-	+	NA	NA	NA	+	-	-	-	+	NA	NA	NA	NA	+	NA	-
**CD10**	-	+	-	-	-	-	+	+	-	-	+	+	-	-	+	-	+	+	-
**ER**	+	+	+	+	+	+	+	NA	-	NA	NA	+	+	-	-	-	+	+	+
**PR**	+	+	+	+	+	+	+	NA	+	NA	NA	+	+	+	NA	-	NA	+	-
**CD99**	+	NA	+	+	-	+	-	+	+	+	NA	+	+	+	NA	+	+	+	+
**HMB45**	-	-	-	-	-	NA	-	NA	-	-	NA	-	NA	NA	NA	NA	-	-	NA
**MelanA**	NA	+	-	NA	-	-	-	-	-	-	-	+	-	-	NA	NA	-	-	NA
**P53**	+	+	NA	+	+	NA	NA	+	NA	NA	NA	+	NA	NA	NA	NA	+	NA	NA
**Ki-67(%)**	2	5	5	10	5	8	3	15	20	10	20	15	20	3	3	40	25	20	15

NA, not available

^*^Excluded from statistics due to molecular translocation suggesting an endometrial stromal neoplasm (JAZF1-SUZ12)

Thirteen cases (13/19, 68.4%) were positive for at least one smooth muscle marker (SMA, Desmin and/or Caldesmon). Tumors were also typically positive for AE1/ AE3, with a positivity rate of 68.4% (13/19). Moreover, fourteen cases (14/19, 73.7%) were positive for at least one hormone receptors (ER, PR). Only one case was truly hormone receptor-negative. As of the 19 cases, 4 were unable to obtain both ER and PR results. Representative images of several typical immunohistochemical markers are shown in Figure S2.

### Tumor microenvironment between benign and malignant cases

**Fig. 2:**
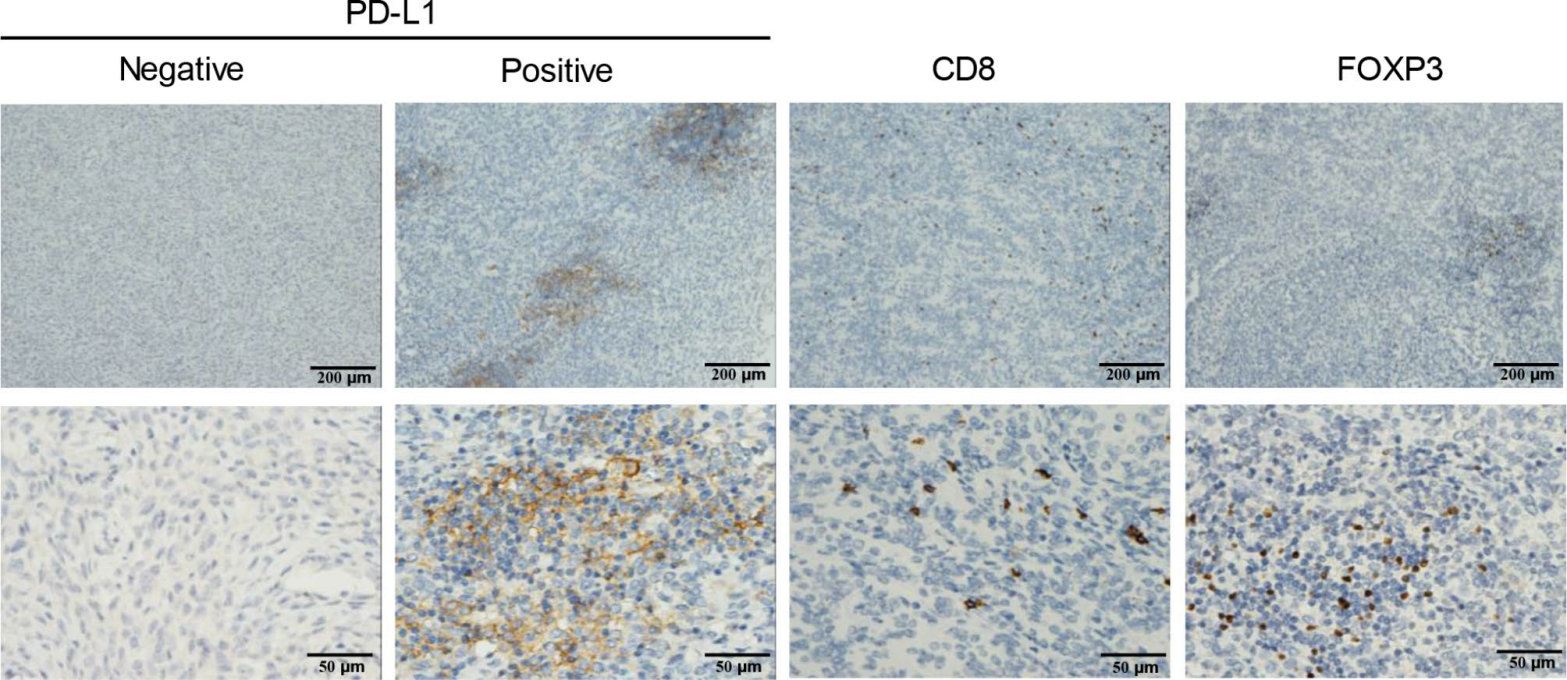
**PD-L1 expression and immune-related cells in tumor.** PD-L1 expression in tumor cells and stromal infiltrated immune cells, CD8 + cells in stromal, and FOXP3 + cells in stromal

**Fig. 3:**
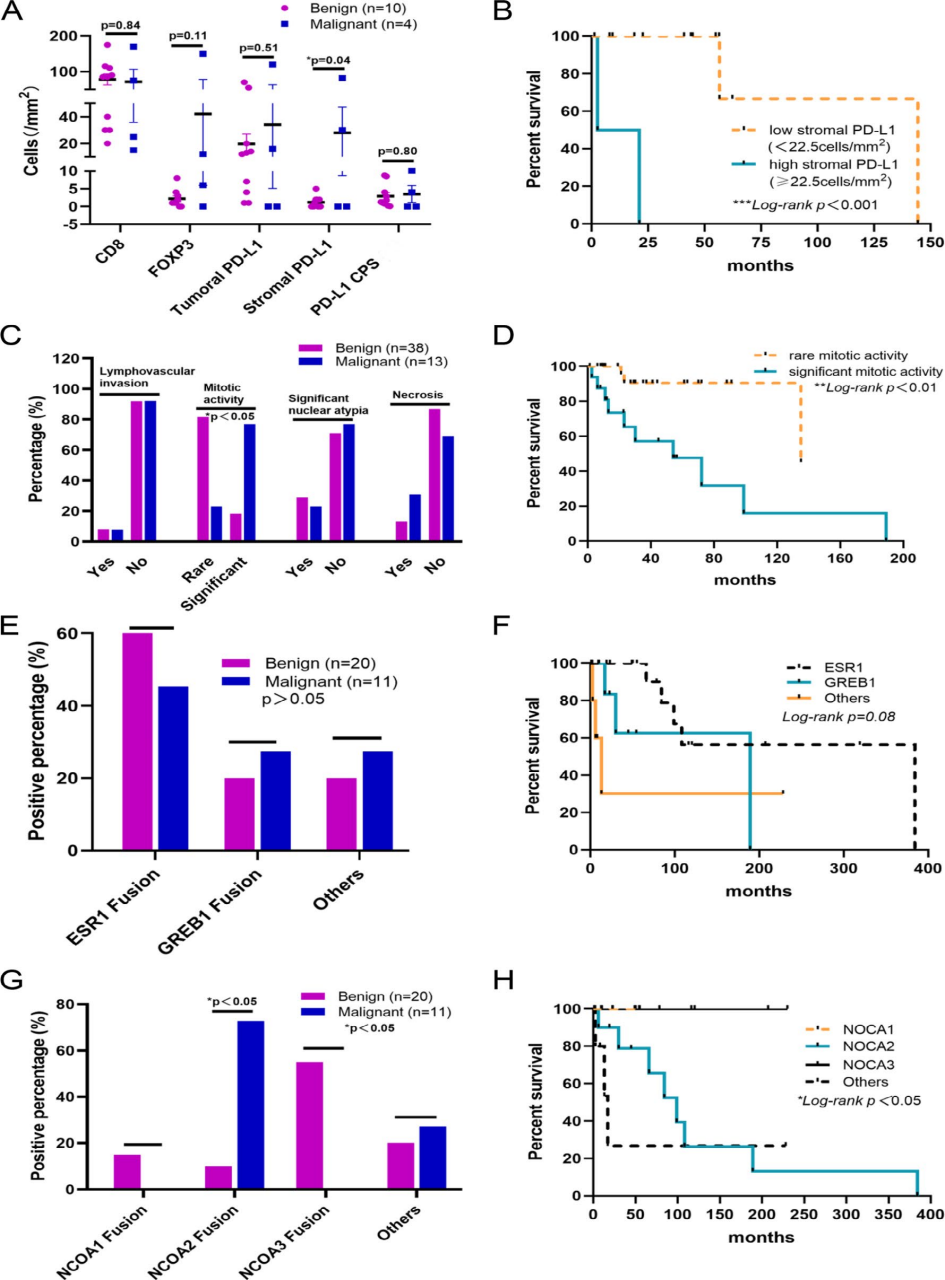
**Immune microenvironment, morphology, and molecular characteristic of the subset of UTROSCT.** A, Immune microenvironment in benign and malignant UTROSCT. B, Predictive factor of stromal PD-L1 for disease-free survival (DFS) by Kappa Meier analysis. C, Morphology in benign and malignant UTROSCT. D, Predictive factor of mitotic activity for DFS by Kappa Meier analysis. E, Gene alteration of 5’ partners in benign and malignant UTROSCT. F, Gene alteration of 5’ partners for DFS by Kappa Meier analysis. G, Gene alteration of 3’ partners in benign and malignant UTROSCT. H, Predictive factor of gene alteration of 3’ partners for DFS by Kappa Meier analysis. *p<0.05, *Log-rank p<0.05

The tumor microenvironment was also assessed in our study, and the recurrence and/or metastasis of UTROSCT was defined as malignant cases. PD-L1, FOXP3, and CD8 expression in either tumor cells or stromal infiltrated immune cells was also analyzed (Fig. 2; Table 3). Although there was no statistical difference between tumoral PD-L1, PD-L1 (CPS), CD8^+^lymphocytes, and FOXP3^+^lymphocytes, the PD-L1 expression in stromal infiltrated immune cells (stromal PD-L1) revealed that malignant cases had markedly higher levels than those that were benign (Fig. 3A). In line with this, disease free survival (DFS) analysis indicated that the high stromal PD-L1 expression group (≥ 22.5 cells/mm^2^) had worse prognoses than those with low stromal PD-L1 expression (Fig. 3B). All immune-related markers between GREB1- and ESR1-rearranged UTROSCTs were also shown in Table 3.

**Table 3:** PD-L1 expression and tumor-associated lymphocytes in UTROSCT

	UTROSCT (n)			
	Benign behavior (n = 10)	Malignant behavior (n = 4)	GREB1-rearranged (n = 2)	ESR1-rearranged (n = 4)
Tumoral PD-L1 (/mm^2^) (mean, range)	38.8(1-260)	34.0(0-120)	35.5(1–70)	6.0(0–12)
Stromal PD-L1 (/mm^2^) (mean, range)	2.7 (0–82)	28.0(0–82)	1.0 (0–2)	1.5(0–5)
PD-L1 (CPS) (mean, range)	6.9 (0.11-52)	3.5 (0–10)	4.4 (0.11–8.75)	0.7(0-1.07)
CD8 + lymphocytes (/mm^2^) (mean, range)	105.4(20–350)	71.3(15–170)	102.5(30–175)	142.5(25–350)
FOXP3 + lymphocytes(/mm^2^) (mean, range)	3.4 (0–10)	42.0(0-150)	4.5 (1–8)	5.0 (3–7)

### Morphologic features between benign and malignant cases

We also compared the morphologic features between benign and malignant cases (Supplementary Table 4). Regrettably, there were no significant differences in features between these two groups, including mean age, mean size of tumor, lymphovascular invasion, mitotic activity, significant nuclear atypia, or necrosis (Supplementary Table 4). However, when we added other cases from the literature that contained these characteristics and a similar follow-up (Supplementary Table 5), the results changed. Although lymphovascular invasion, significant nuclear atypia, and necrosis still had no statistically significant difference, mitotic activity was markedly different between the two groups (Table 4). More specifically, malignant cases were more likely to have mitotic activity than benign cases (Fig. 3C). Consistent with this result, cases with rare mitotic activity had better prognosis in our DFS assessment (Fig. 3D).

**Table 4:** Comparison of findings between benign and malignant cases. (Added cases from literature)

	Mean age (*P* > 0.05)	Mean size of tumor (*P* > 0.05)	Lymphovascular invasion (*P* > 0.05)	Mitotic activity (^***^P<0.001)	Significant nuclear atypia (*P* > 0.05)	Necrosis (*P* > 0.05)
Benign behavior (n = 38)	48.3y	4.8 cm	Yes: 3 (7.9%)	Rare/occasional:31 (81.6%)	Yes: 11 (28.9%)	Yes: 5 (13.2%)
			No: 35 (92.1%)	Significant: 7 (18.4%)	No: 27 (71.1%)	No: 33 (86.8%)
Malignant behavior (n = 13)	51.5y	6.3 cm	Yes: 1 (7.7%)	Rare/occasional: 3 (23.1%)	Yes: 3 (23.1%)	Yes: 4 (30.8%)
			No: 12 (92.3%)	Significant: 10 (76.9%)	No: 10 (76.9%)	No: 9 (69.2%)

### Molecular characteristics between benign and malignant cases

As only two cases in the malignant group had clear gene alterations, we were unable to conduct any statistical analysis. Therefore, we added other cases obtained from the literature to allow for analysis. This included cases that had identified molecular characteristics and similar follow-up; note that we excluded any cases with molecular characteristics that were not available in our already-included 19 cases (Supplementary Table 6). Unfortunately, there was no significant difference between groups when assessing for gene alterations of the 5’ partners including GREB1 and ESR1. (Fig. 3E; Table 5) There was also no significant difference in their respective survival curves (Fig. 3F). However, malignant cases were more likely to have an altered 3’ partner NOCA2 than benign cases (Fig. 3G; Table 5). Our DFS assessment also revealed that patients with a NOCA2 alteration had shorter DFS than those with NCOA1 and NCOA3 alterations (Fig. 3H).

**Table 5:** Comparison of gene alterations between benign and malignant cases. (Added cases from literature)

Gene fusion type		UTROSCT (n = 31)	
		Benign behavior (n = 20)	Malignant behavior (n = 11)
the 3’ partners (^*^P<0.05)	NCOA1 Fusion	3 (15.0%)	0(0.0%)
	NCOA2 Fusion	2 (10.0%)	8(72.7%)
	NCOA3 Fusion	11(55.0%)	0(0.0%)
	Others	4 (20.0%)	3(27.3%)
the 5’ partners (*P* > 0.05)	ESR1 Fusion	12 (60.0%)	5(45.4%)
	GREB1 Fusion	4 (20.0%)	3(27.3%)
	Others	4 (20.0%)	3(27.3%)

**Fig. 4:**
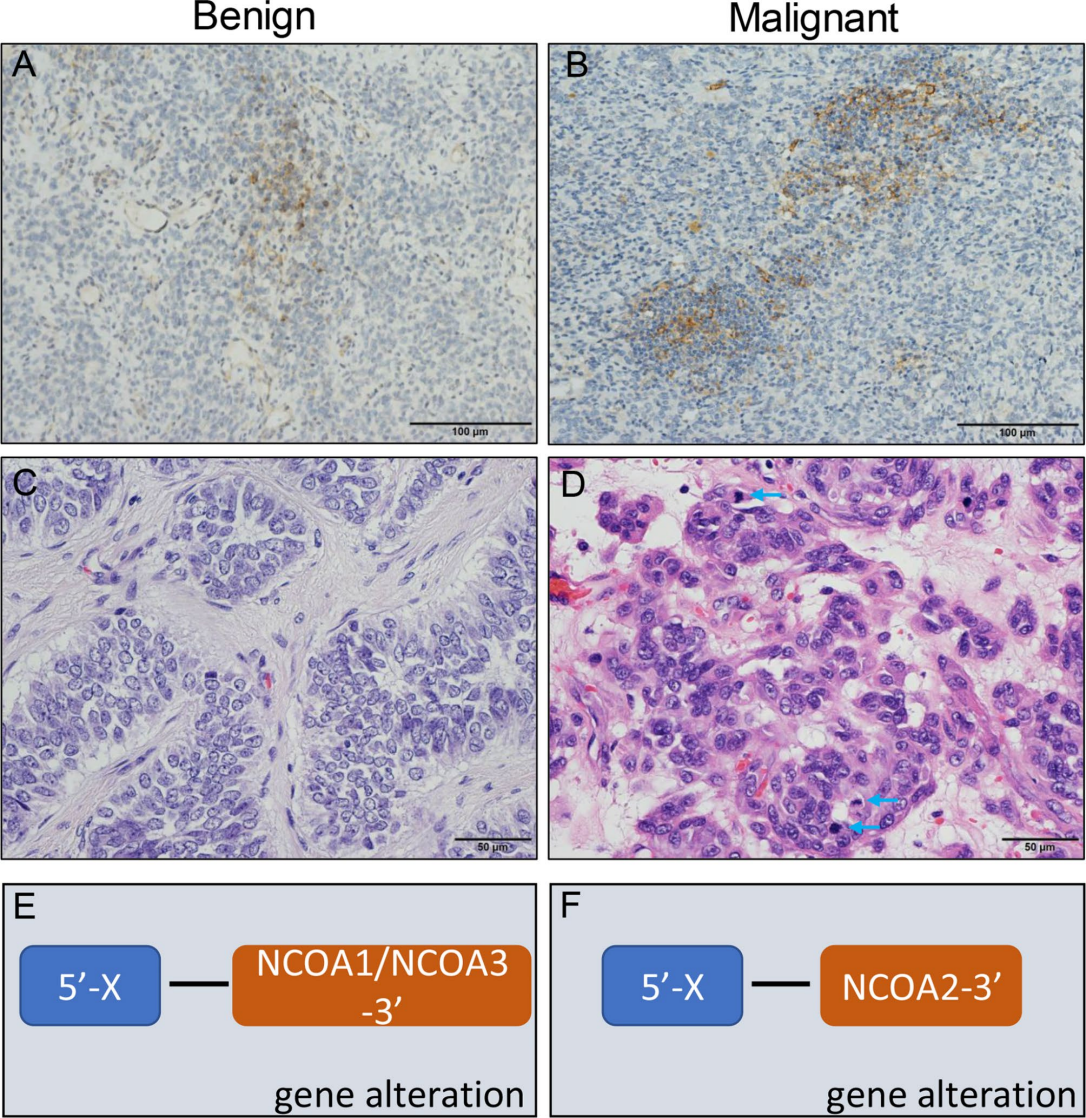
**Representative images of stromal PD-L1 expression, mitotic activity, and gene alteration of the subset of UTROSCT.** Stromal PD-L1 expression in benign UTROSCT (A), and malignant UTROSCT (B). Mitotic activity in benign UTROSCT (C), and malignant UTROSCT (D). Blue arrow: mitotic. Gene alteration in benign UTROSCT (E), and malignant UTROSCT (F)

Moreover, the representative images of stromal PD-L1 and mitotic activity in malignant and benign UTROSCT were shown in Fig. 4, as well as the diagrammatic sketch of gene alteration.

## Discussion

UTROSCT is a tumor with uncertain potential malignancy. It presents with a variety of morphologies, but is mainly characterized by its sexual cord-like differentiation [13]. However, with the increasing number of reported recurrent cases [2, 11, 13, 14], a better understanding of the potential subset of aggressive UTROSCT has become urgent. In our study, the incidence of malignant UTROSCT reached nearly 30%. Given this, we sought to better understand the tumor immune infiltration, histological, and molecular characteristics of aggressive UTROSCT.

We first described and evaluated the morphologic, immunophenotypic, and molecular characteristics of identified cases of UTROSCT. The morphologic and immunophenotypic characteristics of these cases met the diagnostic criteria of UTROSCT as provided by the WHO. The main morphologic types contained corded, trabecular, sertoliform, retiform, and etc. In most of our cases, we identified at least one positive sex cord marker. Unfortunately, one patient was identified with a JAZF1-SUZ12 fusion, suggesting endometrial stromal sarcoma; thus, this patient was excluded from all further analysis.

Infiltration of tumor immune cells has been studied in serious disease across a variety of cancer types, including lung cancer, hepatocellular carcinoma, and breast cancer [15–17]. Moreover, drugs targeting the PD-1/PD-L1 axis have resulted in huge clinical success in melanomas and other cancers across multiple organ systems and tumor types [18–22]. Recently, Elisheva and his colleague reported that when compared with other uterine smooth muscle tumors, leiomyosarcomas exhibited higher PD-L1 expression and cytotoxic T-cell infiltration [12]. In Kim’s study, PD-L1 expression was separately evaluated on tumor cells, stromal tumor-infiltrating lymphocytes, and intraepithelial tumor-infiltrating lymphocytes [23]. Their results showed that high stromal and intraepithelial PD-L1 expression were related to elevated tumor grade of ovarian epithelial cancers [23]. However, given the low incidence of UTROSCT, there is no literature regarding tumor immune cell infiltration in UTROSCT. Given this, we are the first to investigate the PD-L1 expression and tumor immune cell infiltration in UTROSCT. Due to the low expression of PD-L1, we only analyzed PD-L1 expression on tumoral cells and stromal tumor-infiltrating immune cells (stromal PD-L1). Although there was no statistically significant difference in immune-related cells including FOXP3^+^ cells and CD8^+^ cells between benign and malignant UTROSCT, stromal PD-L1 was higher in malignant UTROSCT relative to benign UTROSCT. The lack of difference in immune-related cells may be due to the small number of cases used in this study. Despite this, a difference in stromal PD-L1 expression across the two groups was identified. Consistent with this, we also found that patients with high stromal PD-L1 expression had worse prognoses. Taken together, these results suggest that patients with high stromal PD-L1 expression are more likely to have recurrent tumors. These findings might also imply that benign and aggressive UTROSCT have different tumor microenvironments and different responses to immune therapy. Limitation with the sample size, further study focus on PD-L1 expression in UTROSCT is needed.

It is well known that morphologic characteristics including lymphovascular invasion, mitotic activity, significant nuclear atypia, and necrosis are always associated with malignant behavior. As such, we also evaluated these features in the previous study. It has been reported that necrosis and significant mitotic activity are significantly different between benign and malignant UTROSCT [11]. Unfortunately, in our study, we did not find any significant differences across the two groups. When we added other cases from literature to bolster our small number of identified cases, we noted that malignant UTROSCT was more likely to have significant mitotic activity (P<0.05). Importantly, patients with rare mitotic activity had better prognosis. However, necrosis remained not statistically different across malignant and benign UTROSCT. The difference observed in our study and other cases provided by the literature hints that the morphologic features in UTROSCT need further study. Importantly, that mitotic activity may be a good marker for identifying malignant UTROSCT.

With the continued development of molecular biology techniques, several molecular features of UTROSCT have been recognized including key genes in the sex hormone pathway and (co)activator oncogenes [24–26]. In line with previous work, we also identified these fusion genes, including estrogen receptor 1 (ESR1) and growth regulating estrogen receptor binding 1 (GREB1) as the 5’ partners, and NCOA1-3 as the 3’ partners. In 11 cases of UTROSCT assessed using RNA-seq, ESR1/GREB1-NCOA1/2/3 was observed in 7 cases (7/11, 63.6%). The molecular characteristics of the remaining cases remained unclear. The molecular features of aggressive UTROSCT have been previously described in several case reports. For instance, in Jennifer’s study that featured long-term follow-up, three patients that had an ESR1-NCOA2 gene fusion had recurrent tumors [26]. Other work has shown that patients with a NCOA2 gene alteration also recurred [13, 27]. Due to the limited number of samples included here, we were unable to perform statistical analysis among the gene alterations. When added with other cases obtained from the literature, we were better able to elucidate differences in gene alterations between benign and malignant UTROSCT. These results showed that patients with a NCOA2 alteration were more likely to have aggressive UTROSCT, with correspondingly shorter DFS than patients with NCOA1 and NCOA3 alterations.

In summary, this study offers the first opportunity to systematically assess the morphology, tumor immune infiltration, and molecular characteristic of aggressive UTROSCT. Interestingly, malignant UTROSCT was more likely to have significant mitotic activity, high expression of stromal PD-L1, and NCOA2 gene alteration. In line with these results, our DFS survival assessment showed that those patients with significant mitotic activity, high expression of stromal PD-L1, and NCOA2 gene alteration also had shorter DFS.

## Electronic supplementary material

Below is the link to the electronic supplementary material.

Supplementary Material 1

## Data Availability

The authenticity of this article has been validated by uploading the key raw data onto the Research Data Deposit public platform (www.researchdata.org.cn) with the approval RDD number RDDB2022244517. All data included in this study are available upon request by contact with the corresponding author.
