# Supplementary material for: PD-L1 expression, morphology, and molecular characteristic of a subset of aggressive uterine tumor resembling ovarian sex cord tumor and a literature review
Source: J Ovarian Res. 2023 May 23;16:102. doi: 10.1186/s13048-023-01183-5 (PMC10207776; doi:10.1186/s13048-023-01183-5)
Supplement: Supplementary file 1 — Supplementary Material 1 [file 13048_2023_1183_MOESM1_ESM.docx]

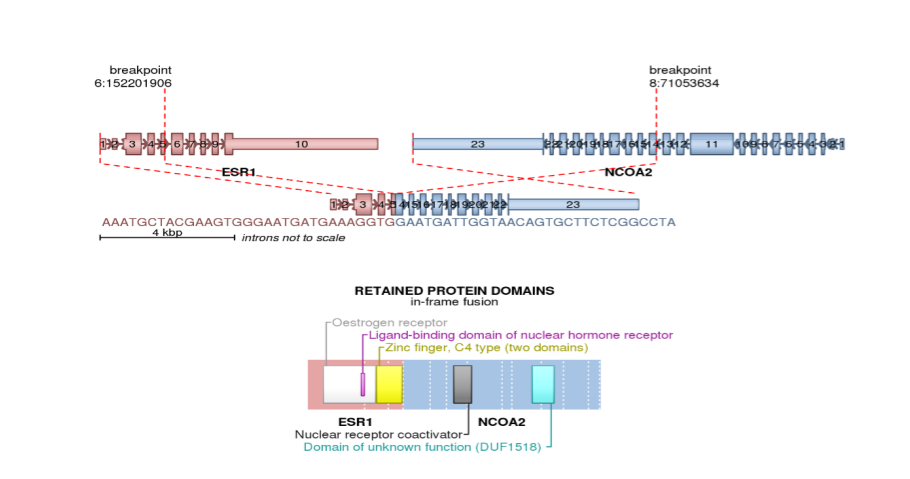


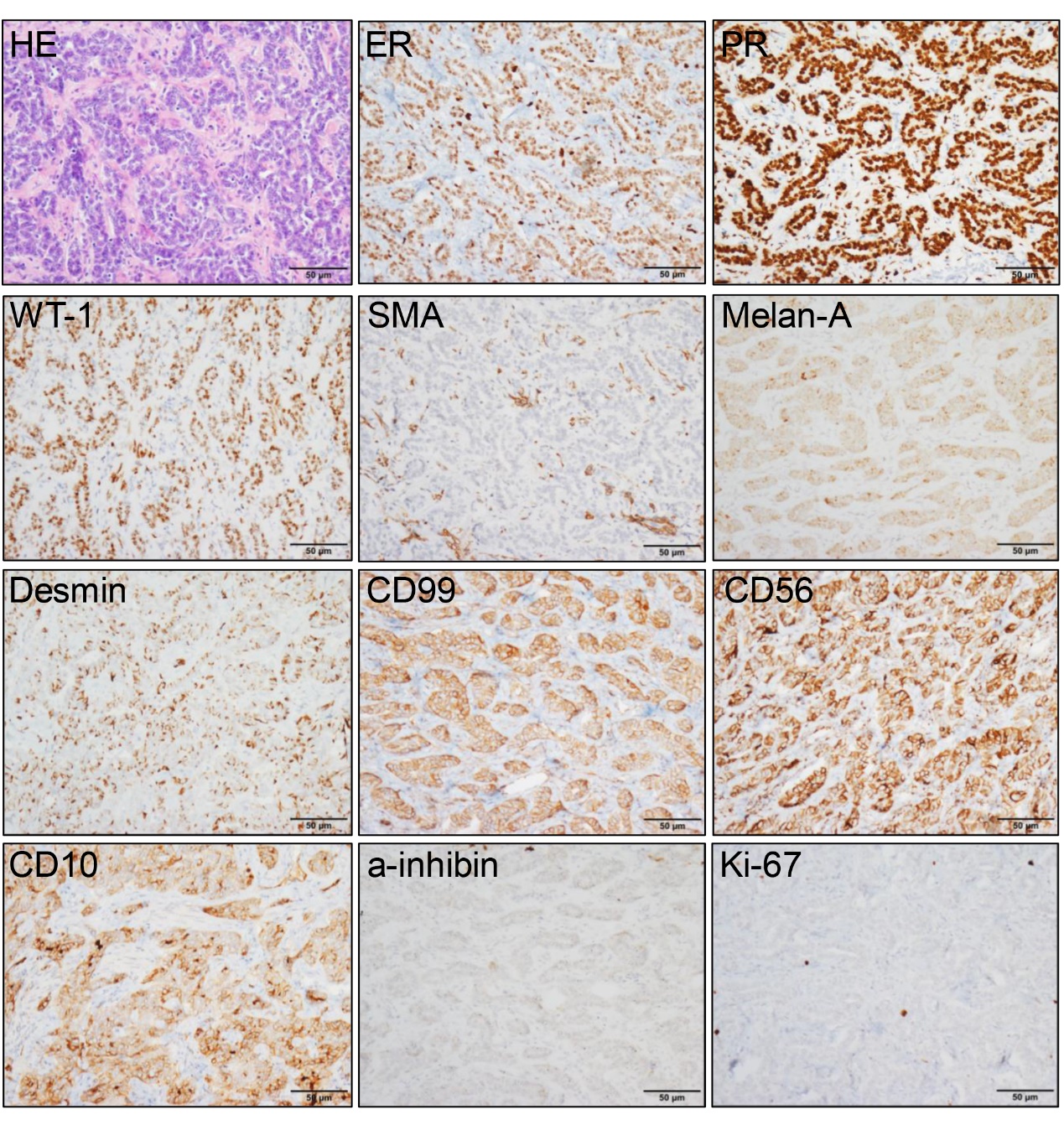
Figure S1. Detailed diagrammatic sketch of RNA sequencing results for Case 4.

Figure S2. Immunohistochemistry showing staining for ER, PR, WT1, SMA, Melan-A, Desmin, CD99, CD56, CD10, a-Inhibin, and Ki67.

**Supplementary Table 1. Detailed information of antibodies.**

| Antibodies | dilution | catalog | company |
| --- | --- | --- | --- |
| Inhibin | 1:100 | MAB-0801 | Maixin Biotech |
| Calretinin | 1:100 | ZA-0026 | ZSGB |
| WT1 | 1:100 | ZA-0269 | ZSGB |
| CD56 | 1:100 | ZM-0057 | ZSGB |
| AE1/AE3 | 1:100 | MAB-0671 | Maixin Biotech |
| Desmin | 1:100 | ZA-0610 | ZSGB |
| SMA | 1:100 | ZM-0003 | ZSGB |
| Caldesmon | 1:100 | ZA-0535 | ZSGB |
| CD10 | 1:100 | MAB-0668 | Maixin Biotech |
| ER | 1:100 | ZM-0104 | ZSGB |
| PR | RTU | 790-2223 | Roche |
| CD99 | 1:100 | ZM-0296 | ZSGB |
| HMB45 | 1:100 | ZM-0187 | ZSGB |
| MelanA | 1:100 | ZM-0398 | ZSGB |
| P53 | 1:100 | ZM-0408 | ZSGB |
| Ki-67 | 1:100 | ZA-0502 | ZSGB |
| PD-L1 | RTU | 743-7066 | Roche |
| CD8 | 1:100 | ZA-0508 | ZSGB |
| FOXP3 | 1:100 | ab20034 | abcam |

**Supplementary Table 2. Clinical details of malignant cases (n=6).**

| Case | Follow-up period (months) | Outcome | Site of metastasis | Oncological therapy administered |
| --- | --- | --- | --- | --- |
| 4 | 144.4 | Developed metatatic disease 98.8 months after initial presentation | Peritoneum | Docetaxel and gemcitabine; letrozole; palbociclib and fulvestrant  Radiotherapy to peritoneum |
| 9 | 26.3 | Developed metatatic disease 2.5 months after initial presentation; died 26.3 months after initial presentation | Pelvis, colon | NA |
| 10 | 69.9 | Developed metatatic disease 54.1 months after initial presentation | NA | Docetaxel and gemcitabine |
| 13 | 56.5 | Developed metatatic disease 13.0 months after initial presentation; developed lung metastasis 13.0 months following initial presentation | Pelvis, lung | NA |
| 14 | 195.3 | Developed metatatic disease 189 months after initial presentation | Pelvis, colon | Letrozole; megestrol; doxorubicine |
| 19 | 21.1 | Developed metatatic disease 14.4 months after initial presentation | Lung | NA |

**NA, not available.**

**Supplementary Table 3. Comparison of GREB1-rearranged uterine tumors versus ESR1-rearranged UTROSCT.**

| Case | Fusion Gene | Age (y) | Tumor size (cm) | Tumor Margins | Lymphovascular invasion | Mitotic activity | Significant nuclear atypia | Necrosis | Follow-up |
| --- | --- | --- | --- | --- | --- | --- | --- | --- | --- |
| **GREB1-rearranged UTROSCT** | | | | | | | | | |
| 5 | GREB1-NCOA2 | 53 | 3 | Infiltrative | No | 2 | No | No | NTR (44.7m) |
| 12 | GREB1-NCOA1 | 58 | 4 | Infiltrative | No | 2 | Yes | No | NTR (22.6m) |
| 14 | GREB1-NCOA2 | 55 | 13 | Infiltrative | No | 2 | Yes | No | Metastasis (195.3m) |
| **ESR1-rearranged UTROSCT** | | | | | | | | | |
| 1 | ESR1-NCOA2 | 31 | 2.5 | Infiltrative | No | <1 | Yes | No | NTR (18.9m) |
| 4 | ESR1-NCOA2 | 41 | 5.5 | Infiltrative | No | 3 | No | No | Metastasis (144.4m) |
| 6 | ESR1-NCOA3 | 33 | 3 | Infiltrative | No | 2 | No | No | NTR (54.9) |
| 15 | ESR1-NCOA3 | 48 | 3 | Infiltrative | No | <1 | No | Yes | NTR (9.2m) |

**NA, not available; NTR, no tumour recurrence.**

**Supplementary Table 4. Comparison of findings between benign and malignant cases.**

|  | Mean age  (*P*>0.05) | Mean size of tumor  (*P*>0.05) | Tumor Margins (*P*>0.05) | Lymphovascular invasion  (*P*>0.05) | Mitotic activity  (*P*>0.05) | Significant nuclear atypia  (*P*>0.05) | Necrosis  (*P*>0.05) |
| --- | --- | --- | --- | --- | --- | --- | --- |
| Benign behavior | 43.0 | 3.0 cm | Yes: 9 (75.0%) | Yes: 0 (0) | Rare/occasional: 5 (41.7%) | Yes: 6 (50.0%) | Yes: 0 (0) |
| (n=12) |  |  | No: 2 (16.7%) | No: 12 (100%) | Significant: 6 (50.0%) | No: 5 (41.7%) | No: 11 (91.7%) |
|  |  |  | NA: 1 (8.3%) |  | NA: 1 (8.3%) | NA: 1 (8.3%) | NA: 1 (8.3%) |
| Malignant behavior | 39.8 | 5.0 cm | Yes: 6 (100%) | Yes: 0 (0) | Rare/occasional: 0 (0) | Yes: 2 (33.3%) | Yes: 3 (50.0%) |
| (n=6) |  |  | No: 0 (0) | No: 6 (100%) | Significant: 5 (83.3%) | No: 3 (50.0%) | No: 2 (33.3%) |
|  |  |  |  |  | NA: 1 (16.7%) | NA: 1 (16.7%) | NA: 1 (16.7%) |

**NA, not available**

**Supplementary Table 5. Morphologic Features of all cases, including our study and literatures.**

| Reference | Case NO. in Reference | age | size | Necrosis | Significant Nuclear atypia | Mitotic activity | Lymph vascular invasion | recurrence | Follow-up  (months) |
| --- | --- | --- | --- | --- | --- | --- | --- | --- | --- |
| Michelle,2017[1] | 1 | 44 | 12.5 | No | No | Yes | No | Yes | 11 |
|  | 2 | 46 | 8 | No | Yes | No | No | No | 11 |
|  | 3 | 75 | NA | No | No | No | No | Yes | 23 |
|  | 4 | 62 | 7 | Yes | No | No | No | Yes | 21 |
|  | 5 | 26 | 2 | No | No | No | No | No | 23 |
|  | 6 | 51 | 3 | No | No | No | No | No | 21 |
|  | 7 | 43 | 2.5 | No | No | No | Yes | No | 19 |
|  | 8 | 54 | 11 | Yes | Yes | No | No | No | 21 |
|  | 9 | 43 | 1 | No | No | Yes | No | Yes | 30 |
|  | 10 | 68 | 9 | No | No | No | No | No | 19 |
|  | 11 | 44 | 1.8 | No | No | No | No | No | 29 |
|  | 12 | 67 | 1.5 | No | Yes | No | Yes | No | 30 |
|  | 13 | 12 | 19.5 | No | No | No | No | No | 27 |
|  | 14 | 56 | 11 | No | No | No | No | No | 35 |
|  | 15 | 54 | 8 | No | No | No | No | No | 37 |
|  | 16 | 42 | 3 | No | No | No | No | No | 41 |
|  | 17 | 47 | 6 | Yes | No | No | No | Yes | 135 |
|  | 18 | 68 | 8 | No | Yes | Yes | No | Yes | 72 |
|  | 19 | 67 | 4.4 | No | No | No | No | No | 61 |
|  | 20 | 36 | 3.5 | No | No | No | No | No | 63 |
|  | 21 | 27 | 1.7 | No | No | No | No | No | 70 |
|  | 22 | 51 | NA | No | No | No | No | No | 135 |
|  | 23 | 86 | 0.4 | No | No | No | No | No | 72 |
|  | 24 | 49 | 3 | No | No | No | No | No | 91 |
|  | 25 | 61 | 12.5 | Yes | No | Yes | Yes | Yes | 6 |
|  | 26 | 54 | 2 | No | No | No | No | No | 88 |
|  | 27 | 71 | 1.7 | No | No | No | No | No | 88 |
|  | 28 | 72 | 7 | No | No | Yes | No | Yes | 23 |
|  | 29 | 58 | 0.8 | No | No | No | No | No | 10 |
|  | 30 | 46 | 2.5 | No | No | No | No | No | 11 |
|  | 31 | 46 | 11 | No | No | No | No | No | 11 |
|  | 32 | 69 | 10 | Yes | Yes | No | No | No | 6 |
|  | 33 | 49 | 18 | No | Yes | No | No | No | 9 |
|  | 34 | 30 | 2 | No | No | No | No | No | 8 |
| Mustafa,2016[2] | 1 | 65 | 8 | Yes | No | Yes | No | No | 12 |
| Our research | 1 | 31 | 2.5 | No | Yes | No | No | No | 18.9 |
|  | 2 | 35 | 3 | No | No | No | No | No | 40.9 |
|  | 3 | 51 | 3 | No | No | No | No | No | 43.9 |
|  | 4 | 41 | 5.5 | No | No | Yes | No | Yes | 98.8 |
|  | 5 | 53 | 3 | No | No | Yes | No | No | 44.7 |
|  | 6 | 33 | 3 | No | No | Yes | No | No | 54.9 |
|  | 7 | 48 | 2 | No | No | Yes | No | No | 56.4 |
|  | 9 | 46 | 2.5 | No | No | Yes | No | Yes | 2.5 |
|  | 10 | 19 | 3 | No | No | Yes | No | Yes | 54.1 |
|  | 12 | 58 | 4 | No | Yes | Yes | No | No | 22.6 |
|  | 13 | 36 | 1.5 | Yes | Yes | Yes | No | Yes | 13.0 |
|  | 14 | 55 | 13 | No | Yes | Yes | No | Yes | 189.0 |
|  | 15 | 48 | 3 | No | Yes | No | No | No | 9.2 |
|  | 16 | 44 | 3 | Yes | Yes | Yes | No | No | 1.2 |
|  | 17 | 29 | 3 | Yes | Yes | Yes | Yes | No | 7.9 |
|  | 18 | 42 | 3.3 | No | Yes | No | No | No | 9.2 |

**Supplementary Table 6.** **Molecular characteristics of all cases, including our study and**

**literatures.**

| Reference | Case NO. in Reference | Recurrence | Gene arrangement type | Follow-up  (months) |
| --- | --- | --- | --- | --- |
| Cheng-Han,2019[3] | 1 | No | ESR1-NCOA3 | 116 |
|  | 2 | No | ESR1-NCOA3 | 49 |
|  | 4 | No | ESR1-NCOA3 | 120 |
| Emily, 2020[4] | 3 | No | ESR1-NCOA3 | 78.4 |
|  | 7 | No | GREB1-NCOA1 | 54.3 |
|  | 16 | Yes | ESR1-NCOA2 | 66 |
|  | 18 | No | ESR1-NCOA3 | 50 |
|  | 19 | No | ESR1-NCOA3 | 319 |
|  | 20 | No | Negative for NOCA1-3 and JAZF1-SUZ12 | 228 |
|  | 21 | No | ESR1-NCOA3 | 23 |
|  | 22 | No | ESR1-NCOA3 | 207.2 |
|  | 23 | No | ESR1-NCOA3 | 2 |
|  | 24 | No | Negative for NOCA1-3 and JAZF1-SUZ12 | 1 |
|  | 26 | No | GREB1-NCOA1 | 10 |
| Jennifer,2020[5] | 1 | Yes | ESR1-NCOA2 | 84 |
|  | 2 | Yes | ESR1-NCOA2 | 108 |
|  | 3 | Yes | ESR1-NCOA2 | 384 |
| Bin,2020[6] | 1 | Yes | GREB1-NCOA2 | 30 |
| Kelly,2021[7] | 1 | Yes | GTF2A1-NCOA2 | 6 |
| Sabrina,2018[8] | 1 | Yes | GREB1-CTNNB1 | 17 |
| Our research | 1 | No | ESR1-NCOA2 | 18.9 |
|  | 4 | Yes | ESR1-NCOA2 | 98.8 |
|  | 5 | No | GREB1-NCOA2 | 44.7 |
|  | 6 | No | ESR1-NCOA3 | 54.9 |
|  | 9 | Yes | Negative for NOCA1-3 and JAZF1-SUZ12 | 2.5 |
|  | 12 | No | GREB1-NCOA1 | 22.6 |
|  | 13 | Yes | Negative for NOCA1-3 and JAZF1-SUZ12 | 13.0 |
|  | 14 | Yes | GREB1-NCOA2 | 189.0 |
|  | 15 | No | ESR1-NCOA3 | 9.2 |
|  | 16 | No | Negative for NOCA1-3 and JAZF1-SUZ12 | 1.2 |
|  | 17 | No | Negative for NOCA1-3 and JAZF1-SUZ12 | 7.9 |

1. Moore M, McCluggage WG: **Uterine tumour resembling ovarian sex cord tumour: first report of a large series with follow-up.** *Histopathology* 2017, **71:**751-759.

2. Ucar MG, Ilhan TT, Gul A, Ugurluoglu C, Celik C: **Uterine Tumour Resembling Ovarian Sex Cord Tumour- A Rare Entity.** *J Clin Diagn Res* 2016, **10:**QD05-QD07.

3. Lee CH, Kao YC, Lee WR, Hsiao YW, Lu TP, Chu CY, Lin YJ, Huang HY, Hsieh TH, Liu YR, et al: **Clinicopathologic Characterization of GREB1-rearranged Uterine Sarcomas With Variable Sex-Cord Differentiation.** *Am J Surg Pathol* 2019, **43:**928-942.

4. Goebel EA, Hernandez Bonilla S, Dong F, Dickson BC, Hoang LN, Hardisson D, Lacambra MD, Lu FI, Fletcher CDM, Crum CP, et al: **Uterine Tumor Resembling Ovarian Sex Cord Tumor (UTROSCT): A Morphologic and Molecular Study of 26 Cases Confirms Recurrent NCOA1-3 Rearrangement.** *Am J Surg Pathol* 2020, **44:**30-42.

5. Bennett JA, Lastra RR, Barroeta JE, Parilla M, Galbo F, Wanjari P, Young RH, Krausz T, Oliva E: **Uterine Tumor Resembling Ovarian Sex Cord Stromal Tumor (UTROSCT): A Series of 3 Cases With Extensive Rhabdoid Differentiation, Malignant Behavior, and ESR1-NCOA2 Fusions.** *Am J Surg Pathol* 2020, **44:**1563-1572.

6. Chang B, Bai Q, Liang L, Ge H, Yao Q: **Recurrent uterine tumors resembling ovarian sex-cord tumors with the growth regulation by estrogen in breast cancer 1-nuclear receptor coactivator 2 fusion gene: a case report and literature review.** *Diagn Pathol* 2020, **15:**110.

7. Devereaux KA, Kertowidjojo E, Natale K, Ewalt MD, Soslow RA, Hodgson A: **GTF2A1-NCOA2-Associated Uterine Tumor Resembling Ovarian Sex Cord Tumor (UTROSCT) Shows Focal Rhabdoid Morphology and Aggressive Behavior.** *Am J Surg Pathol* 2021, **45:**1725-1728.

8. Croce S, Lesluyes T, Delespaul L, Bonhomme B, Perot G, Velasco V, Mayeur L, Rebier F, Ben Rejeb H, Guyon F, et al: **GREB1-CTNNB1 fusion transcript detected by RNA-sequencing in a uterine tumor resembling ovarian sex cord tumor (UTROSCT): A novel CTNNB1 rearrangement.** *Genes Chromosomes Cancer* 2019, **58:**155-163.
